# Idiopathic Inflammatory Myopathy With Normal Creatine Kinase Levels in an Elderly Patient: A Diagnostic Challenge

**DOI:** 10.7759/cureus.100301

**Published:** 2025-12-28

**Authors:** Premsai Chilakuluri, Vaskar Debnath, Ahmed Barakat, Stergios Boussios, Sunayana Sayani

**Affiliations:** 1 Medicine, Medway Maritime Hospital, Gillingham, GBR; 2 Geriatrics, Medway Maritime Hospital, Gillingham, GBR; 3 Oncology, Medway Maritime Hospital, Gillingham, GBR; 4 Medicine, Smt. NHL Municipal Medical College, Ahmedabad, IND

**Keywords:** dermatomyositis, elderly, elderly myositis presentation, idiopathic inflammatory myopathy, mi-2 antibody polymyositis, muscle biopsy in myositis, myositis with normal creatine kinase, polymyositis

## Abstract

Myositis is an inflammatory muscle disorder characterised by progressive weakness, and in elderly patients, diagnosis can be challenging due to symptom overlap with age-related conditions and misleading inflammatory markers. We describe an elderly male with chronic lower limb weakness initially attributed to infection based on elevated inflammatory markers, with persistently normal creatine kinase (CK) levels delaying recognition. Subsequent investigations revealed positive Mi-2 antibodies, an MRI demonstrating bilateral muscle oedema with increased signal intensity, and muscle biopsy findings of HLA class I upregulation with focal complement deposition, consistent with idiopathic inflammatory myopathy. The patient responded well to corticosteroids and mycophenolate mofetil, achieving significant functional recovery. This case underscores the diagnostic difficulties of idiopathic inflammatory myopathy in the elderly, particularly when CK is normal, where initial misattribution to infection led to diagnostic delay and significant functional decline, highlighting the importance of comprehensive evaluation to avoid misdiagnosis and enable timely treatment.

## Introduction

Idiopathic inflammatory myopathies (IIMs) are a heterogeneous group of rare autoimmune muscle disorders characterised by chronic inflammation, progressive weakness, and variable systemic involvement, with an estimated incidence of approximately 0.2-2 cases per 100,000 person-years and prevalence between 2 and 25 per 100,000, varying across geographic regions and study populations [1]. Subtypes include dermatomyositis, polymyositis, antisynthetase syndrome, immune-mediated necrotising myopathy, overlap syndromes, and inclusion body myositis. These subtypes represent a spectrum of disorders that may be classified based on clinical features, autoantibody profiles, and histopathological findings, with overlap syndromes and inclusion body myositis representing distinct entities [2]. Although uncommon, IIMs can significantly impair quality of life and, particularly in dermatomyositis and certain autoantibody-defined subgroups, are associated with an increased risk of malignancy [3].

Diagnosing IIM in elderly patients is particularly challenging, as clinical features often overlap with age-related conditions such as sarcopenia or polymyalgia rheumatica, leading to delayed recognition and misdiagnosis. Furthermore, atypical presentations, such as normal creatine kinase (CK) levels or absence of skin manifestations, may further obscure the diagnosis, as CK can remain normal in chronic low-grade disease, advanced muscle atrophy, or certain inflammatory myopathy subtypes. In such cases, ancillary investigations, including myositis-specific antibodies, magnetic resonance imaging (MRI), and muscle biopsy, are essential to establish the diagnosis and guide appropriate management [4].

We present the case of an elderly male with IIM who exhibited progressive weakness despite persistently normal CK levels. This case highlights the diagnostic difficulties associated with atypical presentations in the elderly and underscores the importance of a comprehensive evaluation incorporating serology, imaging, and histopathology. This article was previously presented as a meeting abstract at the British Geriatrics Society (BGS) North West and Mersey Regional Meeting on July 4, 2024.

## Case presentation

An elderly male presented with a three-month history of progressively worsening fatigue and muscle weakness, which initially began insidiously and gradually progressed to near dependence on a wheelchair by the time of presentation. His wife reported significant functional decline over this period, with increasing difficulty climbing stairs and requiring assistance. His medical history included chronic kidney disease, COVID-19 infection in 2021, and mixed anxiety and depressive disorder. Examination revealed asymmetrical muscle weakness, more pronounced on the left, with reduced muscle bulk in the lower limbs and a myopathic gait. Strength testing demonstrated grade 4/5 power in most muscle groups, with greater weakness in the proximal lower limbs contributing to significant functional impairment. Deep tendon reflexes were preserved, the sensory examination was unremarkable, and there was no focal sensory loss or neuropathic pain. There were no rashes or other features indicative of connective tissue disease.

Laboratory tests revealed leucocytosis (13 × 10⁹/L; reference: 4-11 × 10⁹/L), elevated C-reactive protein (CRP, 210 mg/L; reference: <5 mg/L), and erythrocyte sedimentation rate (ESR, 95 mm/hr; reference: <20 mm/hr). Serology was positive for Epstein-Barr virus (EBV) and cytomegalovirus (CMV) IgG antibodies, consistent with prior exposure and not indicative of active infection or contributing to the clinical presentation. Complement levels, immunoglobulins, protein electrophoresis, and renal and liver function tests were unremarkable. Initial findings of a urinary tract infection (positive dipstick for bacteria) prompted treatment with intravenous antibiotics, reducing the white cell count. However, persistently elevated CRP levels and lack of clinical improvement necessitated further evaluation. A computed tomography (CT) scan of the chest, abdomen, and pelvis was performed to exclude infection or malignancy but yielded no abnormal findings.

**Figure 1:**
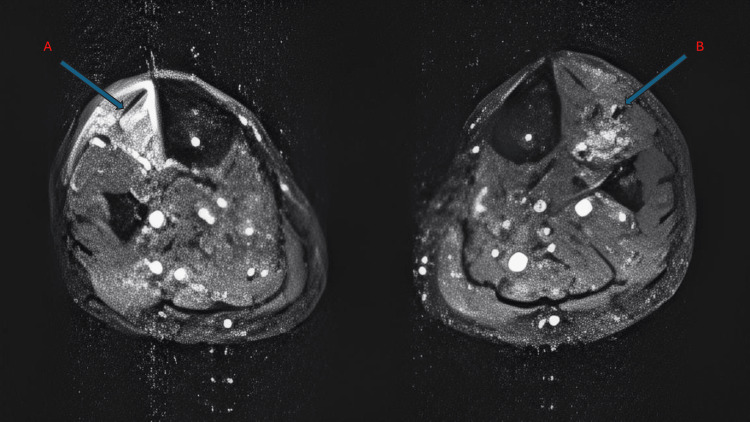
MRI of the lower limbs (A) Cross-section through the left leg showing the tibialis anterior (arrow), with increased signal intensity consistent with oedema and evidence of reduced muscle bulk suggesting early atrophy. (B) Cross-section through the right leg showing the tibialis anterior (arrow), demonstrating hyperintense signal changes compatible with oedema, with less prominent atrophy compared to the left side.

Subsequent autoimmune testing revealed positive Mi-2 antibodies. Notably, this antibody is more commonly associated with dermatomyositis, although the patient did not exhibit any cutaneous features. Due to persistent lower limb weakness, an MRI of the lower limbs was performed, demonstrating bilateral symmetric muscle oedema with increased signal intensity on fluid-sensitive sequences, consistent with active inflammatory changes, particularly involving the anterior compartment muscles (Figure 1). In the context of ongoing weakness despite normal CK levels, these findings supported proceeding to muscle biopsy for definitive diagnosis. Accordingly, a biopsy of the tibialis anterior muscle revealed mild myopathic changes with prominent HLA class I upregulation and focal capillary complement (C5b-9) deposition. There was no evidence of perifascicular atrophy, necrotic fibres, or significant inflammatory infiltrates, supporting an inflammatory myopathy without definitive features of dermatomyositis.

Following consultation with rheumatology, the patient was diagnosed with IIM and commenced on intravenous methylprednisolone 500 mg daily for three days, followed by oral prednisolone at 60 mg daily with appropriate bone and gastric protection. Mycophenolate mofetil 500 mg twice daily was initiated as a steroid-sparing agent, selected in view of the patient's age and comorbid chronic kidney disease, and its favourable tolerability and monitoring profile compared with alternative immunosuppressive agents such as methotrexate or azathioprine. Prior to initiation, baseline infection risk was assessed, with tuberculosis and viral hepatitis screened for and excluded, and there was no evidence of active infection at the time of treatment commencement.

The patient was reviewed two months post-discharge and demonstrated significant clinical improvement, accompanied by a marked reduction in inflammatory markers, including CRP and ESR. Initially bedbound, he regained mobility and could again use stairs independently. Mycophenolate mofetil was continued for maintenance immunosuppression, with a gradual taper of prednisolone toward a low-dose maintenance regimen. Ongoing three-monthly monitoring included full blood count, renal and liver function tests, inflammatory markers, and CK levels to assess disease activity and treatment safety.

## Discussion

The first descriptions of myositis date back to the 19th century, notably by Ernst Wagner, who published works on polymyositis and dermatomyositis in 1863 and 1887 [5]. Contemporary advances in myositis-specific autoantibody profiling, muscle MRI, and refined histopathological classification have since refined the diagnosis of myositis, including atypical presentations in the elderly. Notably, paraneoplastic myositis, particularly in dermatomyositis and selected autoantibody-defined subgroups such as anti-TIF1 gamma and NXP2, can further complicate diagnosis due to overlapping symptoms, and vigilant cancer screening remains essential.

Myositis can present with a range of laboratory abnormalities, but elevated CK levels are typically a hallmark of the disease [6]. However, CK may be normal in subsets of inflammatory myopathies such as inclusion body myositis and, occasionally, polymyositis or dermatomyositis, especially in advanced stages when muscle atrophy is prominent. Additionally, serum aldolase can be elevated even when CK is normal, serving as an alternative biomarker [7]. Serum aldolase was not measured in this patient, which represents a limitation of the biochemical assessment. Our patient's persistently normal CK levels likely reflected a degree of chronicity, supported by imaging findings of reduced muscle bulk with mild proximal atrophy and biopsy features demonstrating subtle myopathic change without prominent necrosis, rather than active widespread muscle breakdown. He scored approximately 9.5 points on the 2017 EULAR ACR classification criteria for IIM, derived from age at onset, objective proximal muscle weakness, myositis-specific autoantibody positivity, and supportive muscle biopsy features, exceeding the threshold for a definite classification. This objective score reinforces the diagnosis despite normal CK levels and the absence of rash.

Table 1 presents all reported cases of myositis with normal CK levels in patients over 60 years.

**Table 1:** Reported cases of myositis with normal CK levels in patients over 60 years

Author	Year	Patient Age	Disease	CK Levels	Muscle Group/Organ Involved
Mackie, Lucy et al. [8]	2024	76	Anti-TIF-1gamma dermatomyositis	Normal	Legs (symmetrical weakness)
Deniz, Rabia et al. [9]	2023	81	Inclusion body myositis (IBM)	Normal	Distal upper, proximal lower extremities
Jevtic, Dorde et al. [10]	2023	67	Seronegative inflammatory myositis evolving to DM	Normal	Proximal lower extremities
Gu, J et al. [11]	2022	75	Anti-OJ antibody-positive anti-synthetase syndrome	Normal	Multiple joints and muscles (biceps, quadriceps)
Kanbayashi, T et al. [12]	2021	79	Myasthenia gravis with inflammatory myopathy	Normal	Generalised muscle weakness
Kwan, C et al. [13]	2020	61	Clinically amyopathic dermatomyositis	Normal	Skin and interstitial lung disease
Lam, Luke et al. [14]	2013	68	Inclusion body myositis (IBM)	Normal	Proximal lower, distal upper extremities
Janerowicz, D et al. [15]	2009	74	Hydroxyurea-induced dermatomyositis-like eruption	Normal	Skin, particularly on the dorsal hand surfaces
Chatterjee, S et al. [16]	2005	62	Rheumatoid vasculitis with myositis	Normal	Proximal muscles
Yoshidome, Y et al. [17]	2007	62	Polymyositis complicated with myasthenic crisis	Normal	Respiratory muscles

Polymyositis vs. dermatomyositis

This case illustrates the diagnostic challenge of distinguishing between IIM subtypes. Mi-2 antibody positivity and complement deposition on biopsy are strongly associated with dermatomyositis; however, our patient lacked cutaneous features, which precluded a definitive diagnosis of dermatomyositis. It is also important to note that myositis-specific autoantibodies may occasionally be detected in elderly individuals without classical clinical phenotypes and, therefore, should always be interpreted in conjunction with clinical features, imaging findings, and histopathology. The treating rheumatology team therefore favoured polymyositis, though the entity of dermatomyositis sine dermatitis (dermatomyositis without skin manifestations) remains a possibility.

Importantly, dermatomyositis is associated with an increased risk of malignancy, particularly adenocarcinomas and lymphomas, with the risk being highest within the first year of onset but persisting for up to five years [18]. This association is strongest in certain autoantibody-defined subgroups, such as anti-TIF1 gamma and anti-NXP2, whereas Mi-2 antibody positivity is not typically associated with an increased malignancy risk. Recognising these distinctions is crucial, as they inform the intensity and duration of cancer surveillance. In our patient, a thorough radiological evaluation excluded malignancy, but ongoing vigilance remains appropriate given his age.

Thus, while the absence of cutaneous manifestations, predominant muscle involvement, and the lack of classic dermatomyositis histopathological features, such as perifascicular atrophy, supported the working diagnosis of polymyositis, which in contemporary practice is recognised to be uncommon and often applied as a pragmatic classification after exclusion of other defined inflammatory myopathy subtypes. The serological and pathological findings suggested overlap with the dermatomyositis spectrum, underscoring the fluidity of classification within IIMs and the importance of malignancy surveillance in this population.

Differential diagnosis

Inclusion body myositis is the most common myopathy in individuals over the age of 50 and can present with asymmetric weakness, often involving the distal muscles of the arms and legs [19]. While our patient demonstrated distal weakness, the absence of inclusion bodies on muscle biopsy ruled out this diagnosis. Rheumatology, after careful consideration, diagnosed him with an atypical presentation of polymyositis due to his chronic history, normal CK levels, and distal lower limb involvement.

The delay in diagnosis was multifactorial. Initially, the patient's symptoms of fatigue and muscle aches were attributed to long COVID, particularly given his continued physical activity, which likely masked early signs of myositis. Given his age and gradual functional decline, sarcopenia was also a potential consideration. Additionally, the fluctuating nature of his symptoms led his GP to consider polymyalgia rheumatica; however, the absence of hallmark features such as predominant shoulder and hip girdle stiffness and the lack of a rapid and sustained response to corticosteroids made this diagnosis less likely. As the patient's condition worsened, a more comprehensive workup was initiated, including antibody testing, MRI, and a muscle biopsy, ultimately confirming myositis.

This case highlights the diagnostic challenges in elderly patients, where temporary symptom relief can obscure underlying diseases, emphasising the need for a high index of suspicion and thorough evaluation when clinical progress stalls or deteriorates. Muscle biopsy remains the gold standard for diagnosing myositis; however, biopsy findings should be interpreted in conjunction with clinical features, serology, and MRI, as sampling variability may occasionally render biopsy findings nondiagnostic or misleading [20].

## Conclusions

This case underscores that IIM can present in elderly patients with normal CK levels and both proximal and distal muscle weakness, posing significant diagnostic challenges. In this population, age-related sarcopenia, comorbid conditions, and overlapping clinical features can contribute to diagnostic delay and potential misclassification. Although antibody and biopsy findings suggested dermatomyositis, the absence of cutaneous manifestations and classic dermatomyositis histopathological features, such as perifascicular atrophy, led clinicians to favour polymyositis as a working diagnosis, highlighting the recognised diagnostic overlap within IIMs. In such atypical cases, early recognition and comprehensive evaluation, particularly through MRI and muscle biopsy interpreted in a clinical context, are essential. Prompt treatment can significantly improve function, mobility, and quality of life while reducing caregiver burden.
